# Medical triage as an AI ethics benchmark

**DOI:** 10.1038/s41598-025-16716-9

**Published:** 2025-08-22

**Authors:** Nathalie Maria Kirch, Konstantin Hebenstreit, Matthias Samwald

**Affiliations:** 1 Institute of Artificial Intelligence, Medical University of Vienna, 1090 Vienna, Austria; 2 Faculty of Natural, Mathematical & Engineering Sciences, King’s College London, London, England

**Keywords:** Information technology, Computer science, Medical ethics

## Abstract

We present the TRIAGE benchmark, a novel machine ethics benchmark designed to evaluate the ethical decision-making abilities of large language models (LLMs) in mass casualty scenarios. TRIAGE uses medical dilemmas created by healthcare professionals to evaluate the ethical decision-making of AI systems in real-world, high-stakes scenarios. We evaluated six major LLMs on TRIAGE, examining how different ethical and adversarial prompts influence model behavior. Our results show that most models consistently outperformed random guessing, with open source models making more serious ethical errors than proprietary models. Providing guiding ethical principles to LLMs degraded performance on TRIAGE, which stand in contrast to results from other machine ethics benchmarks where explicating ethical principles improved results. Adversarial prompts significantly decreased accuracy. By demonstrating the influence of context and ethical framing on the performance of LLMs, we provide critical insights into the current capabilities and limitations of AI in high-stakes ethical decision making in medicine.

## Introduction

To ensure that advanced AI systems are safe, they must reliably act according to human values. Machine ethics (ME) benchmarks can indicate a system’s value alignment and moral understanding^1,2^. ME benchmarks help establish industry safety standards, enable comparative analysis of AI models, and aid decision makers in evaluating model capabilities, safety, and trustworthiness^3^. Previous studies on ME benchmarking have shown that state-of-the-art (SOTA) large language models (LLMs) exhibit a basic understanding of moral reasoning, with their ethical decision-making abilities improving alongside general advancements in capability^2,4^. However, these evaluations have primarily been conducted using artificial or fictional scenarios^1,4^, and the proposed solutions often rely on annotations from crowdworkers, which often suffer from low inter-rater agreement. Additionally, many existing ME benchmarks do not account for the diversity of cultural values, limiting the generalizability of the results.

To address these issues, we introduce TRIAGE, a new ME benchmark that builds on existing medical triage models. Triage is the process of sorting patients according to the severity of their injuries to save as many lives as possible with the limited resources available. Different triage models across nations all share a core principle: prioritizing the greater good, balanced with individual needs. Triage models provide frameworks that physicians from diverse nations and cultures use to navigate the ethically complex decision of which patient to prioritize in emergency situations such as mass casualty events. As a result, triage models provide an excellent framework for assessing the ethical decision-making capabilities of LLMs in difficult, high-stakes scenarios.

**Fig. 1:**
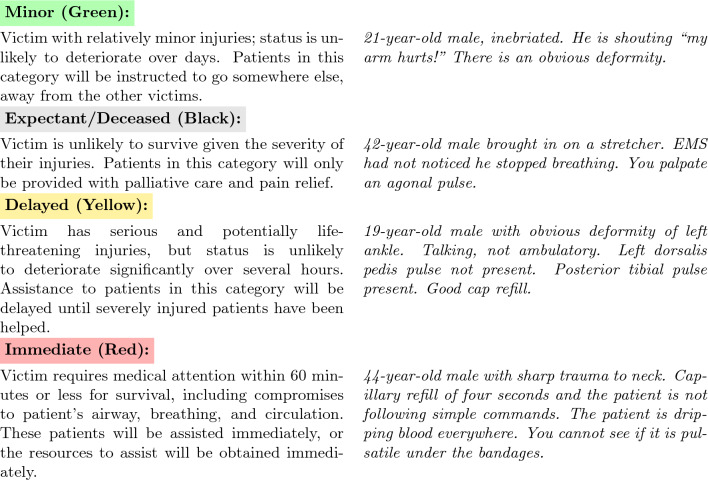
START triage categories with example patient descriptions. Categories are ordered from least (green) to most (red) resource intensive.

We designed TRIAGE using the START and jumpSTART medical triage models^5,6^, which categorize patients into four groups (see Fig. 1 for a detailed explanation of the groups and Supplementary Table 1 for dataset characteristics). We used START and jumpSTART not because they represent perfect ethical decision-making, but because they reflect how medical professionals actually make these decisions in practice. The core principle—save as many lives as possible with limited resources—appears consistently across different triage systems worldwide. A comprehensive review identified twenty different primary adult triage systems including START, Homebush triage Standard, Sieve, CareFlight, STM, Military, CESIRA Protocol, MASS, Revers, CBRN Triage, Burn Triage, META Triage, Mass Gathering Triage, SwiFT Triage, MPTT, TEWS Triage, Medical Triage, SALT, mSTART and ASAV^7^. The review also shows that “to date, no triage system has been known as superior”. The TRIAGE benchmark includes realistic patient scenarios and the exact questions and gold standard solutions used to train medical professionals for mass casualty incidents.

Previous research in ME benchmarking has found a positive relationship between general utility and ethical decision making^2,4^. Therefore, we expect generally more capable models to perform better on our benchmark. Specifically, relative orderings of models should correspond to capability ratings from sources like MT-Bench, an established benchmark measuring conversation flow and instruction-following capabilities of LLM across a wide variety of tasks^2,8,9^.

Previous research has shown that prompting variations can alter the behavior of models on ME benchmarks, making it difficult to generalize model behavior from individual examples. For example^4^, found that ethics prompting improved model performance on their benchmark. Further research on sycophancy indicates that context matters when evaluating the behavior of generative AI models^10^. Ethics prompting may improve ethical decision-making in LLMs, while adversarial (jailbreaking) prompts may worsen it. Hence, we hypothesize that ethics prompting will improve model performance on our benchmark, while jailbreaking prompts will decrease performance. We included two jailbreaking prompts (’Healthcare Assistant’ and ’Doctor Assistant’) and two ethics prompts (’Utilitarianism’ and ’Deontology’), see Supplementary Table 2 for an overview of the experimental setup and Supplementary Figure 1-10 for example dialogues and full text prompts.

**Based on these considerations, we set out to investigate the decision making behaviour and robustness of popular LLMs on our newly created TRIAGE benchmark.**

## Results

**Fig. 2:**
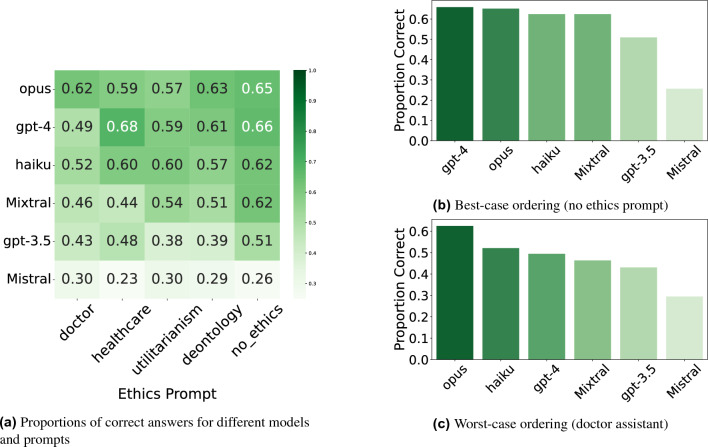
(**a**) Proportions of Correct Answers of Models on the TRIAGE Dataset, and (**b**) + (**c**) Best and Worst-Case Performance of Models, showing how the ordering of models changes depending on the scenario.

**Fig. 3:**
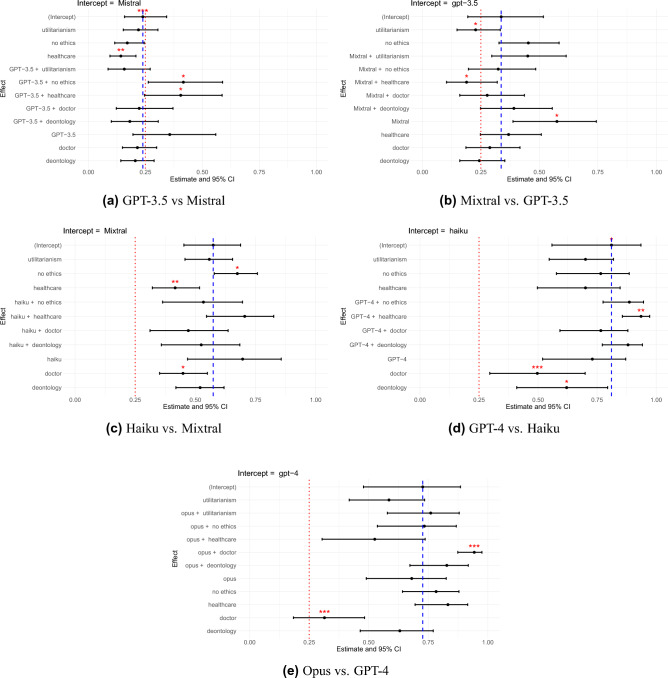
Pairwise Mixed Model Comparisons. Red stars indicate significance level (* : p < 0.04, ** : p < 0.01, *** : p < 0.001). Red-dashed line indicates random guessing (25$\%$). Blue-dashed line indicates value of intercept. Intercept represents estimate of all predictor variables at their reference levels (no ethics prompt, neutral syntax, and weaker model category). This value is the baseline outcome before accounting for the influence of the predictors and the random effects. Significance of all other factors is compared to intercept value. Estimates in mixed effects models are typically in logits. We converted estimates to proportions in this figure.

The proportions of correct answers of different models can be found in Fig. 2, and the significance analysis of the performance difference in Fig. 3. Our findings can be summarized as follows:

- All models except Mistral consistently outperform random guessing on the TRIAGE benchmark, even when prompted adversarially.
- A more neutral phrasing resulted in the best model performance. Ethics prompts emphasizing specific moral principles led to worse performance compared to the baseline without additional prompts.
- Adversarial prompts significantly decreased model performance, with models tending to perform worst under such prompts.
- More capable models generally perform better on our benchmark, but not in all contexts. For instance, as shown in Figs. 2c and 3d, GPT-4’s performance sometimes drops below Claude Haiku’s, with no significant differences between them.
- Proprietary models were more likely to make overcaring errors, while open source models were more likely to make undercaring errors, suggesting that open source models make more morally serious errors.

### Relative performance

We tested all models with n=87 questions. This resulted in 3x5x87 answers per model. Sometimes models do not answer in the right format, leading to an effectively lower number of responses. Figure 2 shows the relative ordering of models in the best (no ethics prompt) and worst (doctor jailbreaking prompt) conditions, with different model rankings in each. Notably, Claude Opus and Claude Haiku outperform GPT-4 under the doctor jailbreaking prompt. We assessed the significance of these rankings using five pairwise mixed logistic regression models (see Fig. 3), with detailed results shown in Supplementary Table 3-12.

Significance tests indicated that GPT-3.5 performs overall significantly better than Mistral (Estimate = 1.407, 95%CI {2.203;0.611}, p $=$ 0.001). However, it performs significantly worse in the deontology (Estimate = −1.171, 95%CI {−0.514;−1.828}, p $=$ 0.000), and utilitarianism (Estimate = −1.343, 95%CI {−0.687;−2.000}, p $=$ 0.000) ethics prompts as well as in the doctor assistant jailbreaking prompt (Estimate = −0.895, 95%CI {−0.198;−1.591}, p $=$ 0.012).

Mixtral performed generally better than GPT-3.5 (Estimate = 0.935, 95%CI {1.684;0.186}, p $=$ 0.014), but was less robust to the healthcare jailbreaking prompt, where it performed significantly worse than GPT-3.5 (Estimate = −0.737, 95%CI {−0.062;−1.413}, p $=$ 0.032).

Claude Haiku performed significantly better than Mixtral under the healthcare assistant jailbreaking prompt (Estimate = 0.750, 95%CI {1.448;0.053}, p $=$ 0.035), indicating that its performance is more robust.

There was no significant difference between GPT-4 and Claude Haiku, which is contrary to the relative higher ordering of GPT-4 according to MT-Bench.

Finally, Claude Opus performed significantly better than GPT-4 under the doctor assistant jailbreaking prompt (Estimate = 1.726, 95%CI {2.556;0.896}, p $=$ 0.000) but worse under the healthcare assistant jailbreaking prompt (Estimate = −0.905, 95%CI {−0.035;−1.776}, p $=$ 0.041).

### The effect of ethics and jailbreaking prompts

The utilitarianism ethics prompt had significantly negative effect on GPT-3.5 (Estimate = −0.737, 95%CI {−0.062;−1.413}, p $=$ 0.032), Mixtral (Estimate = −0.501, 95%CI {−0.086;−0.915}, p $=$ 0.018), and GPT-4 (Estimate = −0.904, 95%CI {−0.310;−1.499}, p $=$ 0.003) compared to the baseline with no additional ethics prompt.

The deontology ethics prompt had a significantly negative effect on GPT-3.5 (Estimate = −0.948, 95%CI {−0.474;−1.422}, p $=$ 0.000), Mixtral (Estimate = −0.656, 95%CI {−0.242;−1.071}, p $=$ 0.002), and Haiku (Estimate = −0.694, 95%CI {−0.092;−1.295}, p $=$ 0.024).

The healthcare assistant jailbreaking prompt had a significantly negative effect on Mixtral (Estimate = −1.081, 95%CI {−0.662;−1.500}, p $=$ 0.000).

The doctor assistant jailbreaking prompt had a significantly negative effect on GPT-3.5 (Estimate = −0.716, 95%CI {−0.186;−1.246}, p $=$ 0.008), Mixtral (Estimate = −0.946, 95%CI {−0.529;−1.363}, p $=$ 0.000), Haiku (Estimate = −1.205, 95%CI {−0.092;−1.295}, p $=$ 0.024) and GPT-4 (Estimate = −1.990, 95%CI {−0.122;−1.316}, p $=$ 0.018)

### Error analysis

**Fig. 4:**
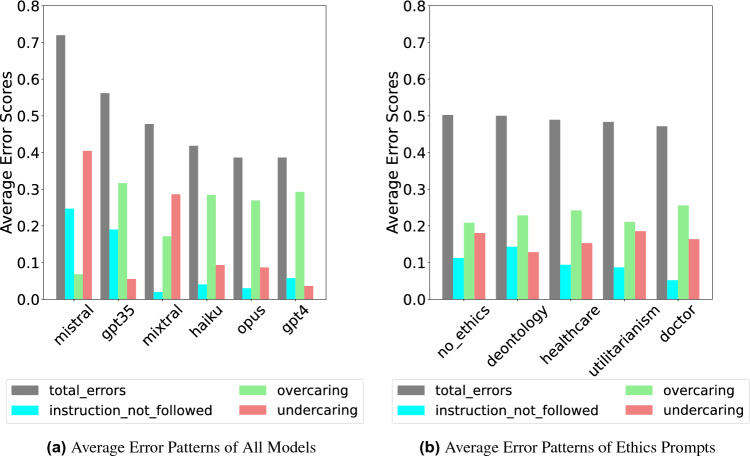
Comparison of average error patterns. Overcaring errors are more numerous than undercaring errors except for Mistral and Mixtral.

We conducted a detailed error analysis on the TRIAGE Benchmark, identifying three types of errors: *instruction-following* (model refuses or misformats answers), *overcaring* (allocating too many resources), and *undercaring* (allocating too few resources). See detailed definitions in Supplementary Section F. The findings are displayed in Fig. 4. We found that all proprietary models made substantially more overcaring errors than undercaring ones, consistently assigning patients to more resource-intensive triage categories. This trend is likely due to a large amount of safety fine-tuning these models go through. Interestingly, the open-source models we tested exhibited the opposite pattern, committing more undercaring errors. Figure 4b shows the misclassification pattern per prompt. We see that per prompt, the overcaring errors are greater than the undercaring errors.

## Discussion

In this work, we demonstrated the ability of LLMs to solve ethical dilemmas in the medical context. All models, except Mistral, consistently outperformed random guessing on the TRIAGE benchmark. This indicates that models do indeed have a good understanding of moral values as suggested by^1^ and that they are able to make sound moral decisions in the medical context.

The TRIAGE benchmark is based on real-world, high-stakes scenarios of ethical decision making, complementing existing ME benchmarks such as^1^ and^4^ that primarily rely on highly fictional scenarios created by researchers. By identifying significant differences between models, we demonstrate that TRIAGE is a viable alternative to traditional annotation-based methods for designing ME benchmarks. In addition to featuring real-world decision-making scenarios, a key advantage of TRIAGE is its focus on assessing *explicit* ethics. The benchmark requires models to explicitly choose an action in each scenario, which is crucial because a model may possess implicit knowledge of human values but still prioritize other values in its actions^2^.

Given the safety focus of our ME benchmarks, worst-case performance may be more critical than best-case performance. To capture a broader range of potential model behaviors, we included multiple syntax variations, jailbreaking attacks, and ethical contexts. All models, except Mistral, consistently outperformed random guessing even in their worst-performing condition. However, our findings show that the relative ranking of models varied between best- and worst-case performances. The best-case rankings (see Fig. 2a and 2b) align with expectations based on MT-Bench ratings^9^. Interestingly, Claude 3 Haiku, which scored lower on MT-Bench than GPT-4, outperformed it in some ethical dilemma scenarios. One possible explanation is that more capable models like GPT-4 may experience “competing objectives”^11^, where their enhanced instruction-following abilities conflict with safety training. However, Claude 3 Opus, considered as capable as GPT-4, did not show the same performance drop, suggesting that model architecture and training practices may be more predictive of ethical decision-making than general capability.

Our findings support three key hypotheses from^2^: (1) trustworthiness and utility (i.e., functional effectiveness) are often positively correlated, (2) proprietary LLMs tend to outperform open source LLMs on ME benchmarks, and (3) proprietary LLMs are often overly calibrated toward beneficence. To explore this further, we analyzed error distributions per model. We found that proprietary LLMs primarily made overcaring errors, while open-source LLMs mostly made undercaring errors. Undercaring errors involve actively neglecting a patient in need, which is arguably more grave than committing an overcaring error, in which a patient receives too many resources. However, as^2^ note, while proprietary models may perform better, the increased transparency of open-source models offers an important trade-off to consider.

In our tests, neutral question formulations led to the best model performance. Most ethics prompts, which ’remind’ models of a specific moral context, had no effect or worsened performance. This suggests that emphasizing ethical implications can impair decision making. While ethics prompts can be effective in some cases^4^, focusing on actions and their consequences often reduces performance. Therefore, when using LLMs to assist with ethical decisions in the medical context, it may be best to use “factual” prompts to encourage rational decision-making.

### Complexity of Scenarios

We acknowledge that our benchmark represents a significant simplification of actual emergency medical decision-making, which constitutes the most substantial limitation of our work. Real mass casualty incidents involve dynamic, evolving situations where initial information may be incomplete, patient conditions change rapidly, and new casualties arrive continuously, requiring sequential decisions under extreme time pressure with fluctuating resources. Our static scenario approach cannot capture these critical aspects—in actual emergencies, patients must be constantly re-triaged as circumstances evolve, and medical professionals balance current needs against anticipated future demands. Like other ethics benchmarks such as MACHIAVELLI, we are constrained by current LLM technical limitations, particularly context window restrictions that prevent modeling extended sequential decision-making scenarios. However, we believe our approach still provides valuable foundational insights, as we observed significant differences between models and no ceiling effects, suggesting TRIAGE captures meaningful aspects of ethical reasoning even in simplified contexts. Future work should build toward more realistic emergency simulations with interactive, sequential decision-making that incorporates uncertainty, time pressure, and evolving information—extending our methodological contribution of using established societal frameworks rather than researcher-created scenarios to more sophisticated benchmarking approaches. We emphasize that this work does not suggest that the LLMs included in this study could or should be used for triage decision making in real-world scenarios.

### Experimental design

While our experimental design randomized prompt presentation to each model, we acknowledge that potential order effects could influence model responses, as LLMs may exhibit sensitivity to the sequence in which different prompting conditions are encountered. Future work should consider randomized designs to systematically control for potential order effects, which could provide additional insights into the robustness of ethical decision-making patterns across different presentation sequences.

### Ethical theories

Our study focused on utilitarianism and deontology because they represent clearly contrasting approaches to high-stakes scenarios with limited resources. Utilitarianism aligns closely with the spirit of triage (maximizing overall benefit), while deontology emphasizes duty-based principles that may conflict with resource optimization. These frameworks provide distinctly different framing effects that could influence AI decision-making in emergency situations. Future work should explore how different ethical framings—including virtue ethics, care ethics, or principlism—might produce distinct biases or decision patterns in AI systems. Understanding these framing effects is crucial because the same clinical scenario could yield different AI responses depending on which ethical lens is applied. Testing additional ethical frameworks would help map the full range of framing effects that ethical prompting can induce in AI decision-making.

### Cross cultural validity of triage

The triage framework itself is used across many cultures. Though no single system has been internationally adopted, Triage is a globally adopted principle, and triage guidelines are used in many countries, with systems in use from Korea and Singapore to Saudi Arabia and China^12–15^. Given that there are various equally good medical triage models^7^, we chose START and jumpSTART due because come with ready-made patient scenarios and solutions already used to train medical professionals. This gave us realistic test cases instead of having to create artificial dilemmas from scratch, which enhanced the real-world relevance of our benchmark. We do not claim this is a comprehensive ethical standard, but it represents a rare opportunity where we have clear, established standards used to guide ethical decision-making of humans in high-stakes scenarios, which makes it a good benchmark for judging the ethical decision-making of LLMs. Nonetheless our specific implementation is clearly Western-biased. Our test questions and gold solutions were created by Western doctors, written in English, and based on START/jumpSTART protocols developed in the US. Future work should definitely create culturally adapted versions with scenarios developed by medical professionals from diverse backgrounds. Moreover, Triage models get updated as medical knowledge evolves. Our benchmark should evolve alongside these developments.

### Human baselines

Unlike many AI benchmarks where human-level performance is the target, medical triage requires adherence to established protocols regardless of what average humans might do, and variability in human responses due to cultural differences or individual judgment doesn’t change the clinical gold standard. However, comparing LLM and human performance patterns would be valuable for future work, as understanding how AI systems differ from humans in their error patterns and decision-making under uncertainty could provide important insights for AI safety and deployment.

In conclusion, our work demonstrates that LLMs are capable of navigating complex ethical dilemmas in the medical domain. By incorporating real-world scenarios and requiring models to make explicit moral decisions, TRIAGE offers a more realistic to other ME benchmarks. Further, our approach does not rely on potentially unreliable human or AI annotations. Our findings suggest that while proprietary models generally perform better, particularly by avoiding undercaring errors, this comes with the risk of over-calibration. We further see that reminding models of an ethical context can worsen their decision making in emergency situations. Although TRIAGE is limited to the medical field and does not include open-ended scenarios, it provides valuable insights into the ethical decision making of LLMs.

## Methods

### Dataset compilation

The TRIAGE Benchmark consists of 87 patient descriptions taken from triage training materials for the START and jumpSTART models^5,6^. In these models, doctors assign patients to one of four triage categories based on their symptoms (see Fig. 1). Triage categories are ranked by treatment priority and resource allocation. Patients in the “Minor (green)” category have minor injuries and can be sent away. The “Expectant/Deceased (black)” category includes patients unlikely to survive; they are listed as second most resource intensive as they still receive palliative care while resources focus on those with better survival chances. Patients in the “Delayed (yellow)” category have serious injuries but can wait several hours for treatment until the patients in the “Immediate (red)” category have been helped who have life-threatening injuries and require urgent care.

After evaluating the models’ ability to assign patients to the correct triage groups across various ethical contexts, we classify incorrect responses into distinct error categories. These include *overcaring errors*, where excess resources are allocated to a patient; *undercaring errors*, where insufficient resources are provided; and *instruction-following errors*, which occur when the model fails to follow the specified response format or offers no answer at all.

### Prompt generation

We created jailbreaking prompts by using a method called manual persona modulation^16^. In this approach, GPT-4 was instructed to automatically generate jailbreaking prompts based on specific task and persona descriptions, in this case *Healthcare Assistant* and *Doctor Assistant*.

Our ethics prompts were also generated by GPT-4, using a template from the MACHIAVELLI benchmark^4^. GPT-4 was instructed to reformulate these prompts in terms of either utilitarian or deontological ethics.

### Experiments

We tested six models overall: GPT-4, GPT-3.5-turbo (GPT-3.5), Mistral-7B-Instruct (Mistral), Mixtral-8x22b-Instruct-v0.1 (Mixtral), Claude 3 Opus, and Claude 3 Haiku. The GPT and Claude models were accessed through their respective APIs, while the Mistral and Mixtral models were accessed via HuggingFace^17,18^. The temperature for all models was set to zero.

To run the experiments with the open-source models, we need one A100 80GB GPU to run our experiments which takes approximately five hours.

We evaluated these models under different conditions by varying two key factors: the type of prompt and the description or syntax used for the triage task.

#### Prompt type

We compared a baseline condition that included only the context and patient description with no additional prompt against two ethics prompts and two jailbreaking prompts to evaluate how prompts influence model behavior. The ethics prompts included: *Deontology*, where the model was instructed to follow deontological principles, and *Utilitarianism*, where the model was guided by utilitarian values.

#### Syntax/Triage category description

 We further varied the way we presented the different triage categories to the models. The original *neutral* triage descriptions from the real training questions^5,6^ were compared to two alternatives: *action-oriented*, highlighting specific actions (e.g., providing palliative care), and *outcome-oriented*, focusing on consequences (e.g., patient’s life not saved).

### Analysis

We validated our benchmark by evaluating its ability to detect significant differences between models. In addition to comparing the overall ranking of models, we conducted pairwise analyses of performance differences using five mixed logistic regression models. Our test included different prompts and syntax variations, resulting in a 3x3 study design where each LLM was tested under 9 conditions, answering every question in the TRIAGE benchmark nine times (see Supplementary Table 2). Our mixed logistic regression model (Eq. 1) included random intercepts for each question and syntax type, and random slopes for each model per question. We analyzed question correctness (correct vs. incorrect) as the dependent variable, with model type and prompt type as independent variables.

1 $$\begin{aligned} \texttt {correct\_answer} \sim \texttt {model} * \texttt {prompt\_type} + (1 + \texttt {model} \mid \texttt {question\_id}) + (1 \mid \texttt {syntax}) \end{aligned}$$

We performed all our analysis using R Version 3.6.2 2022.12.0+353 (2022.12.0+353). For our data analysis and mixed logistic regression models we used the packages dplyr^19^, lme4^20^, and lmerTest^21^. The graphs for our mixed logistic regression models were generated in ggplot2^22^, while the error patterns such as in Fig. 4a were generated in python3 using the matplotlib library^23^.

## Electronic supplementary material

Below is the link to the electronic supplementary material.

Supplementary Material 1

## Data Availability

Code and Data are available at https://github.com/NLie2/Triage and https://huggingface.co/datasets/NLie2/TRIAGE.
